# Attitudes and intentions toward seeking professional psychological help among Chinese healthcare workers during the COVID-19 pandemic

**DOI:** 10.3389/fpsyt.2023.1223895

**Published:** 2023-09-14

**Authors:** Ruirui Huang, Xiaoyan Peng, Shuai Yu, Yumei Tian, Chuanying Gao

**Affiliations:** ^1^School of Nursing, Hunan University of Medicine, Huaihua, China; ^2^Department of Nursing, The First Affiliated Hospital of Hunan University of Medicine, Huaihua, China

**Keywords:** attitudes, intentions, healthcare worker, help-seeking, psychological help

## Abstract

**Background:**

It has been suggested that healthcare workers (HCWs) are experiencing massive stressors that threaten their mental health during the COVID-19 pandemic, but little is known about their attitudes and intentions toward seeking professional psychological help. This study aimed to investigate the attitudes and intentions of Chinese HCWs toward seeking professional psychological help during the COVID-19 pandemic and the associated factors.

**Methods:**

A total of 1,224 Chinese HCWs working in hospitals were recruited online from 12 hospitals in Hunan province in China for a survey conducted in November 2022. The Chinese version of the attitudes toward seeking professional psychological help scale-short form (ATSPPH-SF) and the general help-seeking questionnaire (GHSQ) were separately used to assess the attitudes and intentions of the respondents toward seeking professional psychological help. Demographic and socio-psychological data were collected using a self-developed questionnaire, the perceived social support scale, the self-stigma of seeking help scale, and the patient health questionnaire-9 scale.

**Results:**

The 1,208 HCWs in the final analysis showed relatively negative attitudes and low intentions toward seeking professional psychological help during the COVID-19 pandemic. Results of the multiple linear regression analysis showed that female sex (*p* = 0.031), experience of psychological learning (*p* < 0.001), and social support (*p* < 0.001) had a positive predictive effect on the attitudes of these HCWs toward seeking professional psychological help, whereas self-stigma of seeking help (*p* < 0.001) and depressive symptoms (*p* < 0.001) exerted negative effects. Moreover, experience of psychological learning (*p* = 0.004) and social support (*p* < 0.001) had a positive predictive effect on the intentions of these HCWs toward seeking professional psychological help, whereas divorced marital status (*p* = 0.011) and self-stigma of seeking help (*p* < 0.001) exerted negative effects.

**Conclusion:**

The overall attitudes and intentions of HCWs toward seeking professional psychological help were not optimistic. Effective interventions targeted at influencing factors should be formulated to promote the professional psychological help-seeking attitudes and intentions of HCWs who are at risk of developing mental health problems.

## Introduction

COVID-19 has been a significant global public health problem since late 2019 (1–3). During the COVID-19 pandemic, healthcare workers (HCW) all over the world have experienced massive psychosocial burden and mental health problems (4). A meta-analysis of 65 studies involving 79,437 participants worldwide reported that the overall prevalence rates of anxiety, depression, stress, post-traumatic stress syndrome, insomnia, psychological distress, and burnout among HCW were 34.4%, 31.8%, 40.3%, 11.4%, 27.8%, 46.1%, and 37.4%, respectively, all of which exceeded their corresponding levels before the COVID-19 outbreak (5). A recent survey also showed that 819 (10.5%) of 7,795 frontline Australian HCWs thought about suicide or self-harm during the second wave of the pandemic (6). These data highlight the need to take appropriate measures for addressing COVID-19-related mental health problems.

Since the start of the COVID-19 outbreak, mental health professionals and health authorities in several countries started offering telehealth mental health services, such as telephone hotlines, online 24/7 psychological counselling, synchronous video conferencing, SMS text messaging services, and online psychological self-help interventions, for HCWs and other individuals experiencing psychological crises during the pandemic (7–9). On-site professional psychological services were also provided in designated isolation hospitals. These services were crucial to alleviating the psychological symptoms experienced by HCWs during the crisis (10). However, large-scale studies revealed that only few HCWs actively seek help for their mental problems. For instance, only 2.3% to 18.3% (11–14) of HCWs experiencing mental distress sought professional support, and less than half [388 out of 819 (6)] of those HCWs with thoughts of suicide or self-harm reported professional mental help-seeking behavior during the pandemic.

While the factors associated with the delays, decreases, or deficits in the help-seeking behavior of HCW have been explored in the literature, these studies have mainly focused on demographic (e.g., age and sex), knowledge and structural (e.g., psychological training and time), and social psychological factors (e.g., social support level, depression, anxiety, and stigma) (12, 13). The most commonly reported barriers included confidentiality concerns, lack of time, stigma, lack of awareness about the availability of support, and negative career implications (12, 15, 16). HCWs with previous psychological training experience, high level of social support, depression, and anxiety were more likely to demonstrate mental help-seeking behavior, which can be further promoted by positive work environments and availability of support services; meanwhile, some demographic factors associated with help-seeking behavior remain debated (6, 11, 12, 17).

Psychological help-seeking is an adaptive coping process where individuals seek external assistance from health professionals and others to deal with their mental health problems (18). This process includes three key components, namely, general attitude toward obtaining assistance, future behavioral intentions, and observable behavior. According to theory of planned behavior action, attitudes can strongly influence intentions, which in turn affect actual help-seeking behaviors (19). Therefore, identifying help-seeking attitudes and intentions can help predict actual help-seeking behavior and support those interventions that are aimed toward improving psychological help-seeking. However, previous research on the psychological help-seeking behavior of HCWs has largely ignored their attitudes and intentions toward seeking professional psychological help (20).

While most countries have canceled their strict public health measures over time, China has maintained its long-term “dynamic zero-COVID policy” as an overarching strategy that takes restrictive measures to quickly “zero out” the infected people at the social level and to stop the spread of the virus in a region (21). Accordingly, Chinese HCWs continue to experience massive challenges, psychosocial burden, and suffering in their mental health. The gap between the high prevalence of mental health problems and low rates of actual professional psychological help-seeking behaviors as well as the significant impact of help-seeking attitudes and intentions on actual help-seeking behavior only underscore the significance of investigating the attitudes and intentions of Chinese HCWs toward seeking professional psychological help. Therefore, this paper aims to investigate the level of attitudes and intentions toward seeking professional psychological help among Chinese HCWs who are at risk of experiencing mental health problems during the COVID-19 pandemic and to identify the potential influencing factors associated with such attitudes and intentions. Results of this study can guide the development of targeted interventions and improve the early diagnosis and treatment of HCWs mental health problems.

## Materials and methods

### Study design and participants

A cross-sectional survey was conducted in November 2022 at a time when the participants were experiencing huge psychosocial burden due to the pandemic. The participants were recruited online from 12 hospitals in Hunan Province. These hospitals were selected by convenience sampling. The inclusion criteria for the participants were: aged 18 years or above; HCWs working in hospitals as either doctors or nurses; and were willing to take part in the study voluntarily.

An electronic questionnaire was designed by using a commonly used questionnaire star web/app in China, and an online survey link was initially distributed by the director of the nursing department of the selected hospitals to HCWs through WeChat, which is one of the most commonly used social media applications in China. All participants clicked on the online survey link voluntarily and were informed about the research aims, design, methods, risks, benefits, and how their personal data would be handled. They were also advised that returning the completed questionnaire was equivalent to giving their informed consent. Each participant can only fill out the questionnaire once. The collected data were kept strictly confidential and used only for research purposes. Ethics approval was provided by the medical ethics committee of the Hunan University of Medicine (2022/H120020).

The sample size was determined based on the findings of previous surveys conducted in four European countries (22). With a standard deviation (*σ* = 5.7), permissible error (*δ* = 0.475), and significance level (*ɑ* = 0.05), the required sample size was calculated to be 553 by using an online Chinese sample size calculator. A total of 1,224 HCWs completed the questionnaires, of which 16 questionnaires were discarded for not being filled out by either a doctor or nurse. A total of 1,208 questionnaires were retained for the analysis.

### Measures

#### Socio-demographic characteristics

The sociodemographic characteristics of the participants included their age, sex, marital status, education level, working years, occupation, employment title, department, frontline or non-frontline staff in epidemic prevention and control work, sleeping time, and psychological learning experience.

#### Attitudes toward seeking professional psychological help

The attitudes of HCWs toward seeking professional psychological help was assessed using the Chinese version of the attitudes toward seeking professional psychological help scale-short form (ATSPPH-SF) (23), which comprises 10 items divided into 2 dimensions, namely, openness to seeking treatment for emotional problems and value and need in seeking treatment. Each item was rated on a 4-point Likert scale ranging from 0 (disagree) to 3 (agree). The total scores ranged from 0 to 30, with higher scores indicating more positive attitudes toward seeking professional help. The attitude of a respondent was deemed positive when the total score was ≥20 and when the score for each dimension exceeded 10. Items 2, 4, 8, 9, and 10 were reverse scored. This scale had a Cronbach’s alpha of 0.681, item content validity (I-CVI) of 0.833 to 1.000, and scale content validity index (S-CVI) of 0.932 among community residents in China (23). In this study, ATSPPH-SF obtained a Cronbach’s alpha of 0.651.

#### Intentions toward seeking professional psychological help

The general help-seeking questionnaire (GHSQ) (24) was used to assess the intentions of HCWs to seek help from various sources, such as partners, parents, friends, and mental health professionals. GHSQ can be used as an overall scale that includes all sources of help, or each source can be used as a separate scale. Only one source (mental health professionals) was used in this study to rate the likelihood for HCWs to seek professional psychological help for their mental health problems. The participants were asked a question, “If you were to experience symptoms of depression, how likely would you be to seek psychological help from mental health professionals?” The item was rated on a 7-point Likert scale ranging from 1 (never) to 7 (very likely), with ≥5 indicating possible professional help-seeking intentions and higher scores indicating greater help-seeking intentions. This scale reported a Cronbach’s alpha of 0.85 (24).

#### Social support

Social support was assessed using the perceived social support scale (PSSS) (25), which comprises 12 items divided into 3 dimensions, namely, family support, friends support, and other support. Each item was rated on a 7-point Likert scale ranging from 1 (strongly disagree) to 7 (strongly agree). The total score ranged from 12 to 84, with scores of 12–36 indicating low support, 37–60 indicating moderate support, and 61–84 indicating high support from multiple sources. The Chinese version of the PSSS was validated among Chinese cancer patients, and its coefficients for family support, friend support, other support, and full scale were 0.87, 0.85, 0.91, and 0.88, respectively (26). In this study, PSSS obtained a Cronbach’s alpha of 0.967.

#### Self-stigma of seeking help

The 10-item self-stigma of seeking help scale (SSOSH) was used to measure how much the respondents felt that their self-esteem would be threatened when they seek professional psychological help (27). Each item was rated on a 5-point Likert scale ranging from “strongly disagree” (1) to “strongly agree” (5). Items 2, 4, 5, 7, and 9 were reverse scored. The total score ranged from 10 to 50. High scores indicate greater self-stigma. The Chinese version of the SSOSH was validated among a sample of college students and reported an acceptable internal consistency (Cronbach’s alpha =0.81) (28). In this study, SSOSH reported a Cronbach’s alpha of 0.762.

#### Depression symptoms

The patient health questionnaire-9 (PHQ-9) was used to measure the depressive symptoms felt by the respondents over the past 2 weeks (29). Each item in PHQ-9 was rated on a 4-point Likert scale ranging from 0 (not at all) to 3 (almost every day). The total score ranged from 0 to 27, with scores of 0–4 indicating no depression, 5–9 indicating mild depression, 10–14 indicating moderate depression, 15–19 indicating moderate to severe depression, 20–27 indicating severe depression. The Chinese version of PHQ-9 was validated among patients and reported an acceptable internal consistency (Cronbach’s alpha = 0.851) (30). In this study, PHQ-9 obtained a Cronbach’s alpha of 0.929.

#### Data analysis

All data analyses were performed using SPSS version 22. The characteristics of participants were presented as frequencies or proportions, and the levels of social support, self-stigma, depression symptoms, help-seeking attitudes and intentions were characterized using means and standard deviations. An independent student’s *t*-test was conducted to analyze the statistical differences between different sex, occupation, frontline or non-frontline staff, and psychological learning experience in terms of their professional psychological help-seeking attitudes (the mean ATSPPH-SF sum score) and intentions (the mean GHSQ sum score). Also, one-way ANOVA test were performed to test if the mean ATSPPH-SF sum score and the mean GHSQ sum score differed between the age, marital status, education level, Working years, employment title, department, and sleeping time per day. Pearson correlation analysis was performed to analyze the relationship between professional psychological help-seeking attitudes (ATSPPH-SF sum score) or intentions (GHSQ sum score) and the three continuous variables (social support sum score, self-stigma of seeking help sum score, and depression symptoms sum score). Multiple linear regression models (stepwise) were used to examine the factors that influence professional psychological help-seeking attitudes and intentions. Professional psychological help-seeking attitudes (ATSPPH-SF sum score) or intentions (GHSQ sum score) were analyzed as dependent variable separately and all variables related to professional psychological help-seeking attitudes and intentions in the univariable analysis (*t*-test/one-way ANOVA test) and Pearson correlation analysis were included as independent variables. A *p*-value of <0.05 was considered statistically significant (two-sided test).

## Results

### Characteristics of participants

The 1,208 HCWs participating in this study were aged between 19 and 60 years (*M* = 32.49 years, SD = 7.13 years). The majority of these HCWs were female (*n* = 1,091, 90.3%), 71.1% (*n* = 859) of them were married, and 881 were holding a bachelor’s degree. In addition, 45.1% (*n* = 545) have been working for more than 10 years, and 48.4% (*n* = 585) had junior employment titles. Most of these respondents were nurses (*n* = 1,055, 87.3%) and non-frontline medical workers (*n* = 885, 73.3%), only 26.7% (*n* = 323) were frontline medical workers, and 75.1% (*n* = 907) were working in inpatient departments. Table 1 presents the characteristics of the participants.

**Table 1:** Characteristics of eligible participants (*N* = 1,208).

Sociodemographic characteristics	Frequency	%
*Age (years)*		
19–30	522	43.3
31–40	533	44.1
41–50	125	10.3
>50	27	2.2
*Sex*		
Male	117	9.7
Female	1,091	90.3
*Marital status*		
Married	859	71.1
Unmarried	325	26.9
Divorced	24	2.0
*Education level*		
College diploma and below	290	24.0
Bachelor’s degree	881	72.9
Master’s degree and above	37	3.1
*Working years*		
<3	162	13.4
3–5	165	13.7
5–10	336	27.8
>10	545	45.1
*Occupation*		
Doctor	153	12.7
Nurse	1,055	87.3
*Employment title*		
Junior	585	48.4
Intermediate	522	43.2
Senior/deputy senior	101	8.4
*Department*		
Emergency department	117	9.7
Outpatient department	116	9.6
Inpatient department	907	75.1
Others	68	5.6
*Frontline or non-frontline workers*^a^		
Frontline	323	26.7
Non-frontline	885	73.3
*Sleeping time per day (hours)*		
<6	346	28.6
6–8	792	65.6
>8	70	5.8
*With psychological learning experience*		
Yes	400	33.1
No	808	66.9

a Frontline workers have a high risk of being exposed to COVID-19 patients and body fluids during their epidemic prevention and control work. These workers include those medical staff who are directly involved in the treatment of COVID-19 patients, field nucleic acid collection, COVID-19 fever clinics, or come in contact with the body fluids of COVID-19 patients.

On average, the participants reported moderate to high levels of social support (*M* = 61.04, SD = 13.84), moderate levels of self-stigma of seeking help (*M* = 25.77, SD = 5.20), and mild to moderate levels of depressive (*M* = 7.51, SD = 5.72).

### Attitudes toward seeking professional psychological help and the associated factors

The total scores of ATSPPH-SF ranged from 2 to 30, with a mean score of 18.88 (SD = 4.74), thereby indicating that the participating HCWs had relatively negative help-seeking attitudes.

Table 2 presents the univariable analysis of the characteristic factors related to attitudes toward seeking professional psychological help, whereas Table 3 presents the correlations between the continuous variables and ATSPPH-SF. The statistically significant categorical variables (i.e., sex, sleeping time per day, and psychological learning experience) in Table 2 and continuous variables (i.e., social support, self-stigma, and depression symptoms) in Table 3 were included in the multivariable analysis of attitudes toward seeking professional psychological help. Professional psychological help-seeking attitudes (ATSPPH-SF sum score) was analyzed as dependent variable in the multiple linear regression analysis.

**Table 2:** Univariable analysis characteristics factors related to attitudes and intentions toward seeking professional psychological help.

Characteristics	Help-seeking attitudes			Help-seeking intentions		
	*M* (SD)	*t*/*F*	*p*	*M* (SD)	*t*/*F*	*p*
Age (years)		1.915	0.125		0.746	0.525
19–30	18.91 (4.74)			4.11 (1.67)		
31–40	19.02 (4.60)			4.07 (1.66)		
41–50	18.52 (4.85)			3.86 (1.92)		
>50	16.88 (3.64)			4.04 (1.58)		
Sex		−2.006	**0.045**		0.031	0.975
Male	18.05 (4.75)			4.07 (1.85)		
Female	18.97 (4.73)			4.06 (1.67)		
Marital status		0.193	0.825		3.142	**0.044**
Married	18.89 (4.74)			4.08 (1.70)		
Unmarried	18.90 (4.73)			4.09 (1.64)		
Divorced	18.29 (5.26)			3.21 (1.76)		
Education level		0.442	0.643		0.165	0.848
College degree and below	18.67 (4.51)			4.02 (1.66)		
Bachelor degree	18.96 (4.82)			4.08 (1.70)		
Master degree	18.70 (4.60)			4.14 (1.81)		
Working years		1.854	0.136		0.782	0.504
<3	19.19 (4.48)			4.15 (1.65)		
3–5	18.33 (5.03)			3.99 (1.70)		
5–10	19.25 (4.70)			4.15 (1.62)		
>10	18.73 (4.74)			4.01 (1.74)		
Occupation		0.316	0.752		1.648	0.241
Doctor	19.00 (5.12)			4.27 (1.75)		
Nurse	18.87 (4.69)			4.03 (1.68)		
Employment title		1.850	0.158		0.042	0.959
Junior	18.86 (4.73)			4.06 (1.71)		
Intermediate	18.74 (4.79)			4.06 (1.67)		
Senior/deputy senior	19.73 (4.48)			4.11 (1.69)		
Department		1.695	0.166		0.412	0.744
Emergency department	18.08 (4.38)			3.98 (1.66)		
Outpatient department	19.37 (4.76)			4.22 (1.81)		
Inpatient department	18.90 (4.76)			4.06 (1.68)		
Others	19.22 (5.00)			4.03 (1.71)		
Frontline OR non-frontline staff		0.543	0.587		−0.445	0.657
Frontline	19.00 (4.69)			4.03 (1.66)		
Non-frontline	18.84 (4.76)			4.08 (1.70)		
Sleeping time per day (hours)		9.562	**<0.001**		0.415	0.661
<6	18.07 (4.95)			3.99 (1.75)		
6–8	19.10 (4.57)			4.09 (1.65)		
>8	20.40 (4.96)			4.14 (1.84)		
With psychological learning experience		6.761	**<0.001**		5.235	**<0.001**
Yes	20.17 (4.58)			4.42 (1.68)		
No	18.24 (4.69)			3.89 (1.67)		

*M*, mean; SD, standard deviation; *t*, independent sample *t*-test; *F*, ANOVA-test. Bold = significant predictor.

**Table 3:** Correlations between continuous variables and attitudes or intentions toward seeking professional psychological help.

Variables	Help-seeking attitudes		Help-seeking intentions	
	*r*	*p*	*r*	*p*
Social support	0.377	**<0.001**	0.329	**<0.001**
Self-stigma of seeking help	−0.495	**<0.001**	−0.298	**<0.001**
Depression symptoms	−0.300	**<0.001**	−0.054	0.062

Bold = significant predictor.

Results of the multiple linear regression analysis (Table 4) revealed that having a more positive attitude toward seeking psychological help from professionals was significantly associated with being female (*β* = 0.053, *p* = 0.031), having psychological learning experience (*β* = 0.098, *p* < 0.001), receiving higher social support (*β* = 0.131, *p* < 0.001), having lower self-stigma of seeking help (*β* = −0.370, *p* < 0.001), and having less severe depression (*β* = −0.105, *p* < 0.001). The multiple linear regression model for help-seeking attitudes was statistically significant (*F* = 70.313, *p* < 0.001), with 29.1% of the variance explained by the predictors.

**Table 4:** Multivariable analysis of attitudes toward seeking professional psychological help.

Variables	Help-seeking attitudes				
	*B* (SE)	*β*	*t*	*p*	95% CI
Female	0.848 (0.392)	0.053	2.165	0.031	0.080, 1.617
Male (ref.)	—	—	—	—	—
With psychological learning experience	0.987 (0.251)	0.098	3.932	< 0.001	0.495, 1.480
Without psychological learning experience (ref.)	—	—	—	—	—
Social support	0.045 (0.010)	0.131	4.544	<0.001	0.026, 0.064
Self-stigma	−0.338 (0.027)	−0.370	−12.722	<0.001	−0.390, −0.286
Depression symptoms	−0.087 (0.022)	−0.105	−3.916	<0.001	−0.133, −0.046

Attitudes: *R*^2^ = 0.291, adjusted *R*^2^ = 0.287, *F* = 70.313, *p* < 0.001. *B*, unstandardized coefficients; SE, standard error; *β*, standardized coefficients; 95% CI, 95% confidence interval.

### Intentions toward seeking professional psychological help and the associated factors

The total scores of GHSQ ranged from 1 to 7, with a mean score of 4.06 (SD = 1.69), thereby indicating that the participants had a relatively low intention to seek professional psychological help.

Table 2 presents the univariable analysis of the characteristic factors related to intentions toward seeking professional psychological help, whereas Table 3 presents the correlations between the continuous variables and GHSQ. The statistically significant categorical variables (i.e., marital status and with psychological learning experience) in Table 2 and continuous variables (i.e., social support and self-stigma of seeking help) in Table 3 were included in the multivariable analysis. Professional psychological help-seeking intentions (GHSQ sum score) were analyzed as dependent variable in the multiple linear regression analysis.

The multiple linear regression analysis results in Table 5 show that those participants with greater help-seeking intentions tended to having psychological learning experience (*β* = 0.078, *p* = 0.004), and having higher social support (*β* = 0.229, *p* < 0.001). Having a lower intentions toward seeking psychological help from professionals was significantly associated with divorced marital status (*β* = −0.068, *p* = 0.011) and having lower self-stigma of seeking help (*β* = −0.170, p < 0.001). The regression model for help-seeking intentions was significant (*F* = 39.984, *p* < 0.001), with 14.3% of the variance explained by the predictors.

**Table 5:** Multivariable analysis of intentions toward seeking professional psychological help.

Variables	Help-seeking intentions				
	*B* (SE)	*β*	*t*	*p*	95% CI
Divorced	−0.830 (0.325)	−0.068	−2.551	0.011	−1.468, −0.192
Married (ref.)	—	—	—	—	—
With psychological learning experience	0.280 (0.098)	0.078	2.849	0.004	0.087, 0.473
Without psychological learning experience (ref.)	—	—	—	—	—
Social support	0.028 (0.004)	0.229	7.336	< 0.001	0.021, 0.035
Self-stigma of seeking help	−0.055 (0.010)	−0.170	−5.491	< 0.001	−0.075, −0.036

Intentions: *R*^2^ = 0.143, adjusted *R*^2^ = 0.139, *F* = 39.984, *p* < 0.001. *B*, unstandardized coefficients; SE, standard error; *β*, standardized coefficients; 95% CI, 95% confidence interval.

## Discussion

The main findings of this study were as follows: (1) the mean score of ATSPPH-SF and GHSQ were 18.88 (SD = 4.74) and 4.06 (SD = 1.69) respectively, indicating that Chinese HCWs have a relatively negative attitude and low intention toward seeking professional psychological help for their mental health problems. (2) Being female, having psychological learning experience, and social support were positively associated with the total scores of ATSPPH-SF, whereas self-stigma of seeking help and depression symptoms were negatively associated with the total scores of ATSPPH-SF. (3) Having psychological learning experience and social support were positively associated with the total scores of GHSQ, whereas divorced marital status and self-stigma of seeking help were negatively associated with the total scores of GHSQ.

Chinese HCWs had relatively negative attitudes toward psychological help-seeking from professionals during the COVID-19 pandemic, that is, these workers had a low acceptance of seeking treatment for their mental health problems. The scores of ATSPPH-SF were relatively lower than the those of surveys involving 262 Chinese nurses working in an emergency department before the COVID-19 outbreak (*M* = 21.33, SD = 4.72) (31), patients in the US (*M* = 20.45, SD = 5.51) (32) and the general public in four European countries (*M* = 20.0, SD = 5.7) (22). The low personal help-seeking intentions among Chinese HCWs echoed the findings from other populations (33, 34). These indicated that there are still gaps compared with the more positive help-seeking attitudes and intentions people. Therefore, the attitudes and intentions of HCWs toward seeking professional psychological support should be further improved.

Several influencing factors in the multiple linear regression model were significantly associated with negative attitudes and intentions toward seeking professional psychological help.

Female HCWs held more positive attitudes toward seeking professional psychological help compared with their male counterparts, and this result was consistent with those of studies involving people in the medical profession people and the general public (22, 35–40). Masculinity may limit the ability of men to express their grief (41), and men are more likely to alleviate their pain through alternative solutions, such as alcohol consumption (37). Divorced marital status HCWs had a lower intention toward seeking professional psychological help compared with their married counterparts. A study with analysis of 2,853 cases of psychological assistance hotline help-seekers reported similar findings, that is, the largest number of calls came from married people and fewer (9.7%) from divorced people (42). This may be because divorced people are mainly stressed by work issues, while married people are stressed by both family and work issues, and are more likely to feel anxiety, depression than divorced people (42). Therefore, the need and willingness of divorced people for psychological help are lower than those of married people. Other demographics characteristics, such as age, education level, and occupation, were not associated with the attitudes and intentions of HCWs toward seeking professional psychological help.

Psychological learning experience was positively associated with help-seeking attitudes and intentions, by which an individual’s knowledge/literacy of mental health problems, awareness of mental disorders, and beliefs about effective treatments can be improved (43). Having sufficient knowledge/literacy of mental health problems was also positively associated with help-seeking attitudes and intentions as revealed in other studies focusing on HCWs (38) and other populations (e.g., adolescents and community-dwelling residents) (38, 40, 44). By contrast, having poor knowledge of mental health problems decreases one’s willingness and need to seek professional psychological help (45). However, most HCWs in this study did not report any psychological learning experience. Moreover, the general population has a relatively low mental health literacy due to the lack of psychological education (46, 47). Psychological education about mental illnesses, including their recognition, prevention, and treatment, may influence the help-seeking attitudes and intentions of HCWs who are at risk of developing mental health problems.

Social support positively predicted attitudes, intentions, and help-seeking behavior (12, 48, 49). The participating HCWs reported moderate to high levels of social support from their families, friends, or others. Consistent with the previous literature (48–51), social support from society was positively associated with psychological help-seeking attitudes and intentions. People usually choose their families, friends, or other social networks as their first source of help when facing emotional problems (48, 52). These social networks often play the role of a presenter with lived experience to facilitate the flow of information and advice (53). Individuals interacting with these presenters show less mental-health-related stigma and are more open to seeking treatment (54, 55). Moreover, social support increases the self-efficacy and sleep quality of individuals, which in turn reduce their psychological stress and prompt them to seek help (56). Therefore, social support need to be strengthened to promote people’s positive attitudes and intentions toward seeking mental health help. Social-contact-based interventions can be useful in influencing the psychological help-seeking of HCWs who are at risk of developing mental health problems (54, 55).

The total SSOSH scores of the participating HCWs pointed toward their moderate levels of self-stigma of seeking professional psychological help. Such self-stigma refers to one’s internalization of negative public views toward people who seek professional psychological help, which is one of the most common barriers reported by HCWs who try to seek psychological help (15). Self-stigma has also been strongly associated with the participating HCWs’ negative attitudes and intentions toward receiving psychiatric help in current study. A survey of 8,875 Swiss adults reported similar findings, that is, those people with more negative attitudes toward seeking psychological help also expressed a higher level of self-stigma (40). Another study reported that self-stigma influences the attitudes of college students toward seeking mental health services and is associated with an increased likelihood of having sought mental health services in the past (34). Self-stigma, which is particularly prevalent among HCWs, could instill in them the fear that being diagnosed with a mental health problem would negatively affect their career prospects and highlight their failures in their respective roles (57). The presence of self-stigma decreases the willingness of HCWs and their need to seek psychological help. Therefore, stigma reduction programs (e.g., to offer training that will cause the current stereotypes to change) (58) targeted at HCWs should be organized to encourage positive help-seeking attitudes and intentions, and increase the possibility for these workers to receive mental health treatment.

Mild to moderate levels of depressive was found among the participating HCWs. This physical and mental health indicators was significantly associated with psychological help seeking in the multivariable analysis. Those HCWs with more severe depressive symptoms held more negative attitudes toward seeking professional help, which was consistent with the findings of previous studies focusing on pregnant women and adolescents (35, 48, 59). However, these results also contrasted those of other studies. For instance, one study revealed that public health workers with depression and anxiety were more likely to report actual help-seeking behavior during the COVID-19 pandemic (12), and another study reported that the presence of depression and psychological distress may increase one’s likelihood to seek professional help (60). Such differences can be ascribed to abnormalities in behavioral activation (BAS) and behavioral inhibition (BIS) systems, in which depressed people are more likely to face behavioral inhibition (61) and are more introverted, pessimistic, evasive, and less likely to maintain social interaction compared with non-depressed people (13, 60, 62). If these people are not severely depressed, then they are more likely to rely on themselves to cope with their psychological problems (63) and become willing to seek help. Moreover, having severe symptoms of anxiety, depression, or prior mental health issues can encourage people to utilize the available professional support (13).

### Strengths and limitations

To the best of the authors’ knowledge, this study is the first to investigate the professional psychological help-seeking attitudes, intentions, and associated factors of a large sample of Chinese HCWs during the COVID-19 pandemic. The results of this study could guide mental health providers in developing targeted interventions that can improve the attitudes and intentions of HCWs toward seeking psychological help.

This study also has several limitations that should be acknowledged. First, this study adopts a cross-sectional survey, which makes it impossible to infer causal pathways among attitudes and intentions toward seeking professional help and their influencing factors. Second, nurses comprised 87.3% of the sample, and most of the participating HCWs were female. Therefore, doctors or other HCWs and males were underrepresented in this study.

### Implications

This study offers significant implications for improving the attitudes and intentions of HCWs toward seeking professional psychological help. Chinese HCWs who are at risk of developing mental health problems held relatively negative attitudes and had a low intention to seek professional psychological help, which largely contrasted the high prevalence of mental health problems among this population during the COVID-19 pandemic. The more positive their attitudes and intentions toward seeking help, the more likely these HCWs will demonstrate help-seeking behavior, which in turn can alleviate their work pressure and improve their mental health (19). Therefore, the attitudes and intentions of HCWs toward seeking professional psychological support should be improved.

The significantly positive impact of social support on the help-seeking attitudes and intentions of HCWs highlights the importance of strengthening the role of informal psychological support sources in encouraging HCWs to disclose their feelings, promote their utilization of mental health services, and improve their self-belief in successfully demonstrating a professional psychological help seeking behavior with the desired result. The significantly negative impact of self-stigma on the help-seeking attitudes and intentions of HCWs implies that stigma reduction programs should be developed to reduce the self-stigma associated with mental illness and professional psychological help-seeking behavior. The participating HCWs in this study with depressive symptoms were less willing to seek psychological help from professionals. Therefore, the early recognition of at-risk HCWs, early treatment of their depressive symptoms, and their timely referral to psychological professionals are all critical. Psychological learning experience also has a positive impact on help-seeking attitudes and intentions. Therefore, HCWs should be given essential training to instill in them the knowledge, attitudes, and skills that would lead to positive changes in their help-seeking process.

## Conclusion

The attitudes and intentions of Chinese HCWs toward seeking professional psychological help during the COVID-19 pandemic are relatively negative and low. Positive factors affecting such attitude include being female, having psychological learning experience, and having better social support. Meanwhile, those factors that positively affect these HCWs’ intentions toward seeking psychological help include having psychological learning experience and having better social support. The negative factors affecting attitudes include self-stigma of seeking help and higher level of depression. The negative factors affecting intentions include divorced marital status and self-stigma of seeking help. Interventions that target these factors should be designed to enhance the professional psychological help-seeking attitudes and intentions of HCWs.

## Data availability statement

The raw data supporting the conclusions of this article will be made available by the authors, without undue reservation.

## Ethics statement

The studies involving humans were approved by the Medical Ethics Committee of the Hunan University of Medicine, in Hunan Province, China. The studies were conducted in accordance with the local legislation and institutional requirements. The participants provided their written informed consent to participate in this study.

## Author contributions

RH, XP, SY, and CG conceived, planned, and designed the study. RH and SY wrote the original draft of the manuscript, analyzed the data, and revised the manuscript under the supervision of CG and XP. CG and XP administrated the project, interpreted the data, and oversaw the writing of the paper. RH, SY, and YT performed this experiment, supervised the execution of the study, and checked the quality of data. All authors contributed to the article and approved the submitted version.

## Funding

This work was supported by the Hunan Provincial Philosophy and Social Sciences Program of China (22YBX024), the 2021 Innovation and Entrepreneurship Training Program for college students in Hunan Province (4297), and Science and Technology Innovation platform Program of Huaihua City, Hunan Province, China (2020N2401).

## Conflict of interest

The authors declare that the research was conducted in the absence of any commercial or financial relationships that could be construed as a potential conflict of interest.

## Publisher’s note

All claims expressed in this article are solely those of the authors and do not necessarily represent those of their affiliated organizations, or those of the publisher, the editors and the reviewers. Any product that may be evaluated in this article, or claim that may be made by its manufacturer, is not guaranteed or endorsed by the publisher.
